# The non-linear association between serum iron and severe impairment of activities of daily living in ischemic stroke patients

**DOI:** 10.3389/fneur.2025.1700381

**Published:** 2026-01-15

**Authors:** Ranran Bi, Yupeng Shi, Zhongcheng Xie, Xiaochen Liu, Zhenchao Ma, Fang Cui

**Affiliations:** 1 Department of Rehabilitation Medicine, Shanghai East Hospital, School of Medicine, Tongji University, Shanghai, China; 2 Department of Rehabilitation, Shenzhen Second People's Hospital, The First Affiliated Hospital of Shenzhen University, Shenzhen, China

**Keywords:** activities of daily living, non-linear association, serum iron, stroke, threshold effect

## Abstract

**Objectives:**

This study aims to explore the dose–response relationship and threshold effect of serum iron levels on severe impairment of activities of daily living (ADL) in ischemic stroke patients.

**Methods:**

This cross-sectional study included 2,035 ischemic stroke patients admitted to Shanghai East Hospital from 2020 to 2022. Serum iron levels were measured upon admission, and ADL was evaluated using the Barthel Index. Restricted cubic spline regression, multivariate logistic models, and subgroup analysis were employed to analyze the dose–response relationship.

**Results:**

A non-linear relationship (*p* = 0.005) was observed between serum iron and severe ADL impairment, with an inflection point at 17.5 μmol/L. Below this threshold, each 1 μmol/L increase in serum iron was associated with 9% lower odds of severe ADL impairment (OR = 0.91, 95% CI: 0.876–0.946). No significant association was observed above 17.5 μmol/L (*p* > 0.05). Subgroup analyses revealed no significant interactions in any subgroup.

**Conclusion:**

The study found a non-linear relationship between serum iron and severe ADL impairment after ischemic stroke, with an inflection point at about 17.5 μmol/L. Future prospective studies are necessary to clarify this association.

## Introduction

Stroke ranks as the second leading cause of death worldwide and is a major contributor to disability, with its prevalence increasing, especially in developing nations (1–4). According to the China Stroke Surveillance Report 2021, an estimated 17.8 million adults in China had a stroke in 2020, with 2.2 million leading to disability (5). The majority of strokes are ischemic, resulting from arterial blockage (6). Stroke-related long-term disability is primarily the result of impaired motor function (7, 8). Survivors of stroke frequently experience challenges with daily activities such as bathing and dressing, along with a lower quality of life and diminished participation in community activities (9–11).

Iron is a crucial trace element involved in numerous cellular processes, including oxygen transport, energy metabolism, and antioxidant defense (12). Disruptions in iron metabolism, whether due to deficiency or overload, can lead to adverse health effects, especially in older adults (13). Emerging evidence links iron metabolism to cognitive function, dementia risk, and the progression of neurodegenerative diseases (14, 15), which could indirectly impact the development of activities of daily living (ADL). Several studies have investigated the relationship between iron levels and functional outcomes following ischemic stroke (16). For example, one study suggested that iron stores, measured by serum ferritin levels, may influence prognosis in patients with acute stroke (17). This indicates that iron status may have an indirect effect on ADL by influencing functional recovery after stroke.

In summary, although there is evidence linking iron status, cognitive function, neurodegenerative diseases, and functional outcomes after ischemic stroke, no studies have directly examined the relationship between serum iron levels and ADL post-stroke. This study aims to investigate the dose–response relationship and the threshold effect of serum iron levels on severe ADL impairment in ischemic stroke patients.

## Materials and methods

### Study design and population

**Figure 1:**
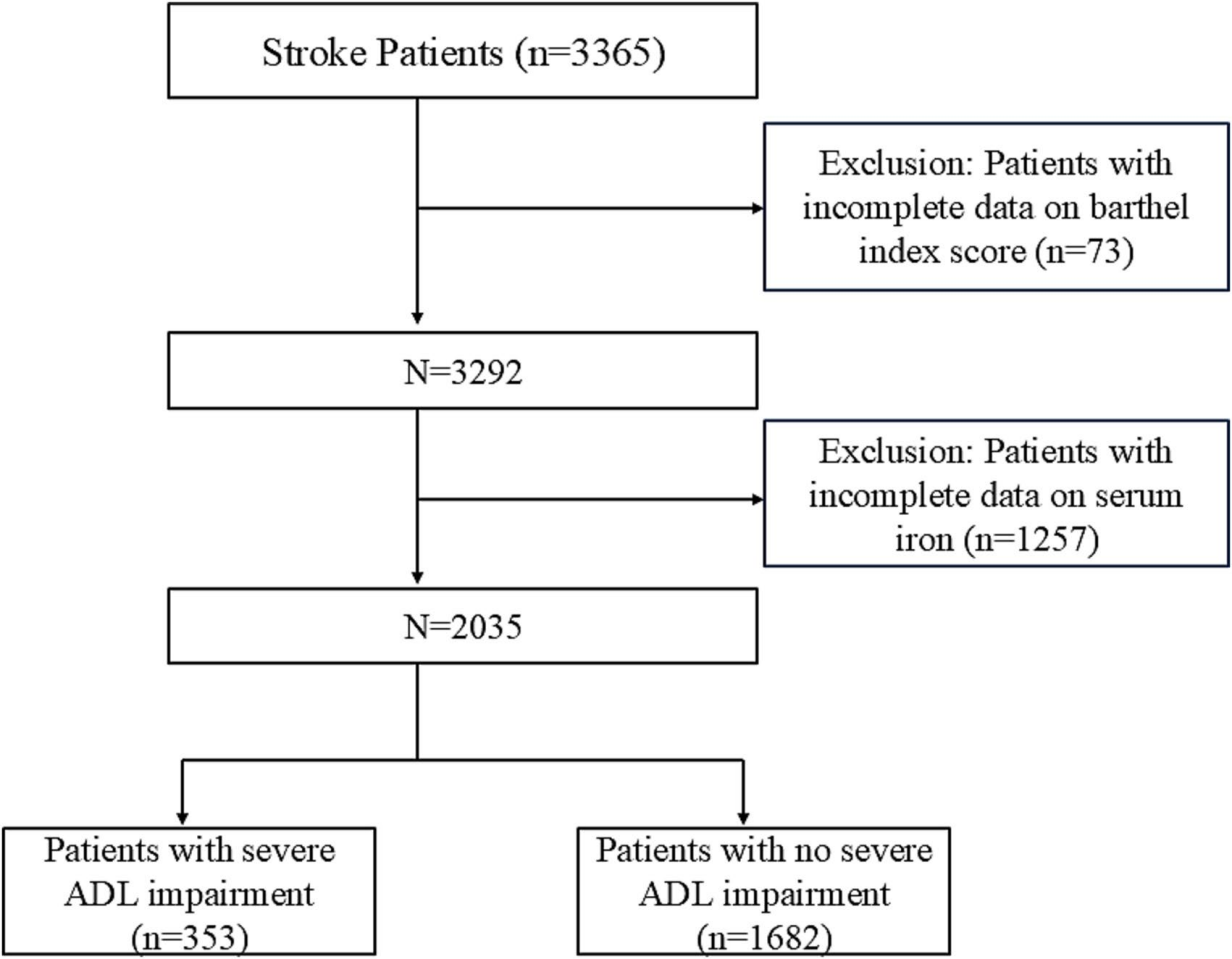
Flowchart of patient selection.

We designed a cross-sectional study of stroke patients admitted to Shanghai East Hospital between January 2020 and August 2022, which was conducted. The study included patients aged 18 years or older, with ischemic stroke diagnoses confirmed within 24 h of admission by cranial CT or MRI. Patients lacking an admission Barthel Index score or without recorded serum iron levels were excluded from the study. The patient selection process is illustrated in Figure 1. This study adhered to the Strengthening the Reporting of Observational Studies in Epidemiology (STROBE) guidelines and received approval from the Ethics Committee of Shanghai East Hospital (No. 2024055). As a retrospective study using anonymized data, informed consent was not necessary. All methods and procedures adhered to the ethical standards set by the World Medical Association’s Declaration of Helsinki on Human Experimentation.

### General data collection

Overall, data collection depends on a combination of prior literature reports and clinical experience (18–20). Demographic and lifestyle data, including height, weight, gender, and age, were collected during the initial hospital admission and entered into the electronic medical records. Height and weight were measured according to World Health Organization standards, and body mass index (BMI) was calculated as weight (kg) divided by height squared (m^2^). Smoking and drinking status were categorized as “yes or no, in the current or past.” Hypertension was diagnosed either by documented history or by blood pressure readings ≥140/90 mmHg on at least two occasions in the hospital. Additional medical history, including diabetes, coronary artery disease, and a history of stroke, was also obtained from the records.

The severity of stroke at admission was assessed using the National Institutes of Health Stroke Scale (NIHSS) (21, 22). The TOAST criteria classified ischemic strokes into five subtypes: small-vessel occlusion, large-artery atherosclerosis, cardio embolism, stroke of other known causes, and stroke of undetermined etiology (23). Additionally, ALT, CRP, and ferritin levels were measured using an automated analyzer by the enzymatic rate method, immunoturbidimetric method, and chemiluminescent method, respectively. Serum iron levels were measured using a Roche analyzer by the colorimetric method within 24 h of the stroke onset. All blood indicators were measured in the morning following an overnight fast.

Activities of daily living (ADLs) refer to the fundamental tasks necessary for self-care and maintaining personal well-being, including personal care, mobility, and eating. The Barthel Index (BI) is a widely recognized tool for evaluating a patient’s ability to carry out basic ADLs. First introduced in 1965 by Dorothea Barthel and Florence Mahoney in the United States, the index assesses 10 functional areas, including feeding, bathing, dressing, toileting, mobility, walking, stair climbing, and bowel and bladder control (24, 25). The total BI score ranges from 0 to 100, with higher scores reflecting greater independence in daily activities. The Barthel Index is extensively applied in assessing functional status across various medical conditions, particularly in studies on stroke, spinal cord injury, dementia, and Parkinson’s disease (26–29). In China, several studies have also employed the BI to evaluate ADL capacity in stroke patients (2). This study assessed the BI score of patients on the first day of admission.

A Barthel Index score below 40 indicates that a patient lacks independence in mobility, self-feeding, personal grooming, and sphincter control (30). Patients are unable to perform daily tasks independently and require assistance from others to complete them. This level of dependence is classified as severe impairment in activities of daily living.

### Statistical analysis

Descriptive analyses were performed for each patient. Categorical variables were presented as frequencies (percentages), while continuous variables were reported as medians (interquartile ranges) or means ± standard deviations, depending on their distribution. Logistic regression models were used to estimate odds ratios (ORs) and 95% confidence intervals (CIs) to assess the association between serum iron levels and severe ADL impairment among ischemic stroke patients. Multiple imputation was used to handle missing data. In Model 1, adjustments were made for age, sex, BMI, and smoking and drinking status. Model 2 included additional adjustments for hypertension, diabetes, coronary heart disease, history of stroke, TOAST classification, and NIHSS score. Model 3 incorporated all variables from Model 2, with further adjustments for Alanine Aminotransferase (ALT), C-Reactive Protein (CRP), and ferritin.

Due to missing data, multiple imputation was performed across the entire study population, and multivariable regression analysis was conducted on the imputed datasets. To assess the robustness of our findings, a sensitivity analysis was performed using a BI score of less than 60 as the cutoff for defining ADL dependence. Since the study did not include a prior statistical power analysis, the sample size was determined based solely on the available data. Additionally, in the sensitivity analysis, the E-value method was used to assess the potential impact of unmeasured confounding on the results.

After adjusting for the variables in Model 3 across the entire study population, we used restricted cubic spline (RCS) regression to examine the nonlinear relationship between serum iron levels and severe ADL impairment, as well as to assess the dose–response association between serum iron and ADL impairment. To investigate the threshold effect of serum iron on severe ADL impairment following ischemic stroke, we applied a smoothed binary logistic regression model. Additionally, likelihood ratio tests and bootstrap regression methods were used to identify significant inflection points in this relationship.

In addition, we examined several variables that could influence the relationship between serum iron levels and severe ADL impairment following ischemic stroke. The variables analyzed included: sex; age (<60 years vs. ≥60 years); body mass index (BMI) categories (<25, 25–29.9, and ≥30 kg/m^2^); smoking and drinking status (yes or no); hypertension (yes or no); and diabetes (yes or no). Multivariate logistic regression was used to assess subgroup heterogeneity, and the likelihood ratio test was employed to explore interactions between subgroups and serum iron levels.

All analyses were performed using R version 4.3.1 (http://www.R-project.org, The R Foundation) and Free Statistics version 1.8. A descriptive study was conducted for each participant, and a two-tailed *p*-value of <0.05 was considered statistically significant.

## Results

### Study population

The study initially enrolled 3,365 patients with ischemic stroke. However, 73 patients were excluded due to missing data on activities of daily living (ADL), and an additional 1,257 patients were excluded because of incomplete serum iron level data. As a result, this cross-sectional study included a total of 2,035 ischemic stroke patients from Shanghai East Hospital, with data collected between 2020 and 2022. Figure 1 provides a detailed overview of the selection process, outlining both inclusion and exclusion criteria.

### Baseline characteristics

Table 1 summarizes the baseline characteristics of the subjects, grouped by quartiles of serum iron. The mean age of the patients was 69.3 ± 11.7 years, with 1,326 (65.2%) being male. Patients with higher serum iron levels were younger, predominantly male, and had a slightly higher BMI. They also have higher BI scores. These patients were more likely to have a history of hypertension. According to the TOAST classification, they were more frequently classified under large-artery atherosclerosis and small-vessel occlusion. In Supplementary Table S1 and Supplementary Table S2, we compare the baseline characteristics of the included and excluded patients, clearly presenting the information regarding missing data in this study.

**Table 1:** The baseline characteristics by categories of serum iron.

Variables	Total	Q1 (<10.3 μmol/L)	Q2 (10.3–13.9 μmol/L)	Q3 (13.9–18.0 μmol/L)	Q4 (≥18.0 μmol/L)	*p*-value
No.	2035	504	513	508	510	
Sex, *n* (%)						< 0.001
Male	1,326 (65.2)	265 (52.6)	305 (59.5)	350 (68.9)	406 (79.6)	
Female	709 (34.8)	239 (47.4)	208 (40.5)	158 (31.1)	104 (20.4)	
Age, Mean ± SD	69.3 ± 11.7	73.4 ± 11.2	69.5 ± 11.5	68.1 ± 11.1	66.1 ± 11.8	< 0.001
BMI, Mean ± SD	24.5 ± 3.4	24.1 ± 3.8	24.5 ± 3.5	24.3 ± 3.3	25.1 ± 3.1	< 0.001
Smoking status, *n* (%)						< 0.001
No	1,088 (58.5)	316 (67.7)	292 (63.5)	263 (55.8)	217 (47)	
Yes	772 (41.5)	151 (32.3)	168 (36.5)	208 (44.2)	245 (53)	
Drinking status, *n* (%)						0.096
No	1,637 (83.9)	413 (85.9)	421 (85.7)	405 (83.5)	398 (80.7)	
Yes	313 (16.1)	68 (14.1)	70 (14.3)	80 (16.5)	95 (19.3)	
Hypertension, *n* (%)						0.134
No	398 (19.9)	89 (18.5)	88 (17.3)	106 (21)	115 (22.7)	
Yes	1,603 (80.1)	391 (81.5)	421 (82.7)	399 (79)	392 (77.3)	
Diabetes, *n* (%)						0.076
No	1,062 (53.1)	242 (50.4)	263 (51.7)	263 (52.1)	294 (58)	
Yes	939 (46.9)	238 (49.6)	246 (48.3)	242 (47.9)	213 (42)	
Coronary heart disease, *n* (%)						0.078
No	1,683 (84.1)	391 (81.5)	422 (82.9)	428 (84.8)	442 (87.2)	
Yes	318 (15.9)	89 (18.5)	87 (17.1)	77 (15.2)	65 (12.8)	
History of stroke, *n* (%)						0.237
No	1,643 (82.1)	389 (81)	408 (80.2)	416 (82.4)	430 (84.8)	
Yes	358 (17.9)	91 (19)	101 (19.8)	89 (17.6)	77 (15.2)	
NIHSS, Median (IQR)	2.0 (1.0, 5.0)	3.0 (2.0, 7.0)	2.0 (1.0, 4.5)	2.0 (1.0, 4.0)	2.0 (1.0, 4.0)	< 0.001
TOAST, *n* (%)						< 0.001
Large-artery atherosclerosis	715 (40.2)	198 (47)	190 (40.8)	160 (35.9)	167 (37.4)	
Small-vessel occlusion	736 (41.3)	112 (26.6)	199 (42.7)	210 (47.1)	215 (48.1)	
Cardio embolism	174 (9.8)	62 (14.7)	44 (9.4)	38 (8.5)	30 (6.7)	
Stroke of another determined etiology	42 (2.4)	12 (2.9)	8 (1.7)	10 (2.2)	12 (2.7)	
Stroke of undetermined etiology	113 (6.3)	37 (8.8)	25 (5.4)	28 (6.3)	23 (5.1)	
Barthel Index Score, Mean± SD	63.0 ± 26.3	51.7 ± 27.8	62.0 ± 25.2	67.7 ± 24.2	70.6 ± 24.0	< 0.001
ALT, Median (IQR)	15.0 (11.0, 22.0)	14.0 (9.0, 20.0)	15.0 (10.2, 20.8)	15.0 (11.0, 23.0)	16.0 (12.0, 24.0)	< 0.001
CRP, Median (IQR)	3.1 (1.6, 11.3)	7.9 (2.2, 30.0)	2.9 (1.6, 9.0)	2.5 (1.6, 6.9)	1.9 (1.6, 6.3)	< 0.001
Ferritin, Median (IQR)	228.0 (138.0, 362.0)	233.0 (128.0, 392.0)	211.0 (122.5, 344.5)	231.5 (147.0, 354.5)	238.0 (140.0, 389.0)	0.154

Q, quartiles; OR, odds ratio; CI, confidence interval; Ref, reference; SD, standard deviation; BMI, Body Mass Index; NIHSS, National Institute of Health stroke scale; TOAST, Trial of ORG 10172 in Acute Stroke Treatment.

After adjusting for confounding factors, multivariate analysis revealed that for every 5 μmol/L increase in serum iron levels, there was 18% lower odds of severe ADL impairment following ischemic stroke. When serum iron levels were examined by quartiles, a negative association with severe ADL impairment was observed after accounting for relevant variables. Compared to patients with lower serum iron levels (<10.3 μmol/L), the adjusted odds ratios (OR) for severe ADL impairment in the second quartile (Q2: 10.3–13.9 μmol/L), third quartile (Q3: 13.9–18.0 μmol/L), and fourth quartile (Q4: >18.0 μmol/L) were 0.68 (95% confidence interval [CI]: 0.47–1), 0.43 (95% CI: 0.28 ~ 0.66), and 0.54 (95% CI: 0.35–0.83), respectively (Table 2).

**Table 2:** Association between serum iron and severe impairment of ADL in ischemic stroke patients (ADL grouped by 40 scores).

Variable	*n*. total	*n*. event_%	Crude model		Model 1		Model 2		Model 3	
			OR (95% CIs)	*p* value	OR (95% CIs)	*p* value	OR (95% CIs)	*p* value	OR (95% CIs)	*p* value
Serum iron (5 μmol/L)	2035	353 (17.3)	0.59 (0.53 ~ 0.67)	<0.001	0.66 (0.59 ~ 0.75)	<0.001	0.76 (0.67 ~ 0.87)	<0.001	0.82 (0.72 ~ 0.94)	0.004
Serum iron group (μmol/L)										
Q1 (<10.3)	504	167 (33.1)	1 (Ref)		1 (Ref)		1 (Ref)		1 (Ref)	
Q2 (10.3–13.9)	513	84 (16.4)	0.40 (0.29 ~ 0.53)	<0.001	0.45 (0.33 ~ 0.61)	<0.001	0.56 (0.39 ~ 0.8)	0.002	0.68 (0.47 ~ 1)	0.049
Q3 (13.9–18.0)	508	51 (10)	0.23 (0.16 ~ 0.32)	<0.001	0.28 (0.2 ~ 0.4)	<0.001	0.36 (0.24 ~ 0.54)	<0.001	0.43 (0.28 ~ 0.66)	<0.001
Q4 (≥18.0)	510	51 (10)	0.22 (0.16 ~ 0.32)	<0.001	0.3 (0.21 ~ 0.43)	<0.001	0.44 (0.29 ~ 0.66)	<0.001	0.54 (0.35 ~ 0.83)	0.005
*p* for trend				<0.001		<0.001		<0.001		<0.001

Q, quartiles; OR, odds ratio; CI, confidence interval; Ref, reference.

Crude model: No adjustment.

Model 1 was adjusted for sex, age, BMI, smoking status, and drinking status.

Model 2 was adjusted for sex, age, BMI, smoking status, drinking status, hypertension, diabetes, coronary heart disease, history of stroke, NIHSS, and TOAST.

Model 3 was adjusted for sex, age, BMI, smoking status, drinking status, hypertension, diabetes, coronary heart disease, history of stroke, NIHSS, TOAST, ALT, CRP, and ferritin.

**Figure 2:**
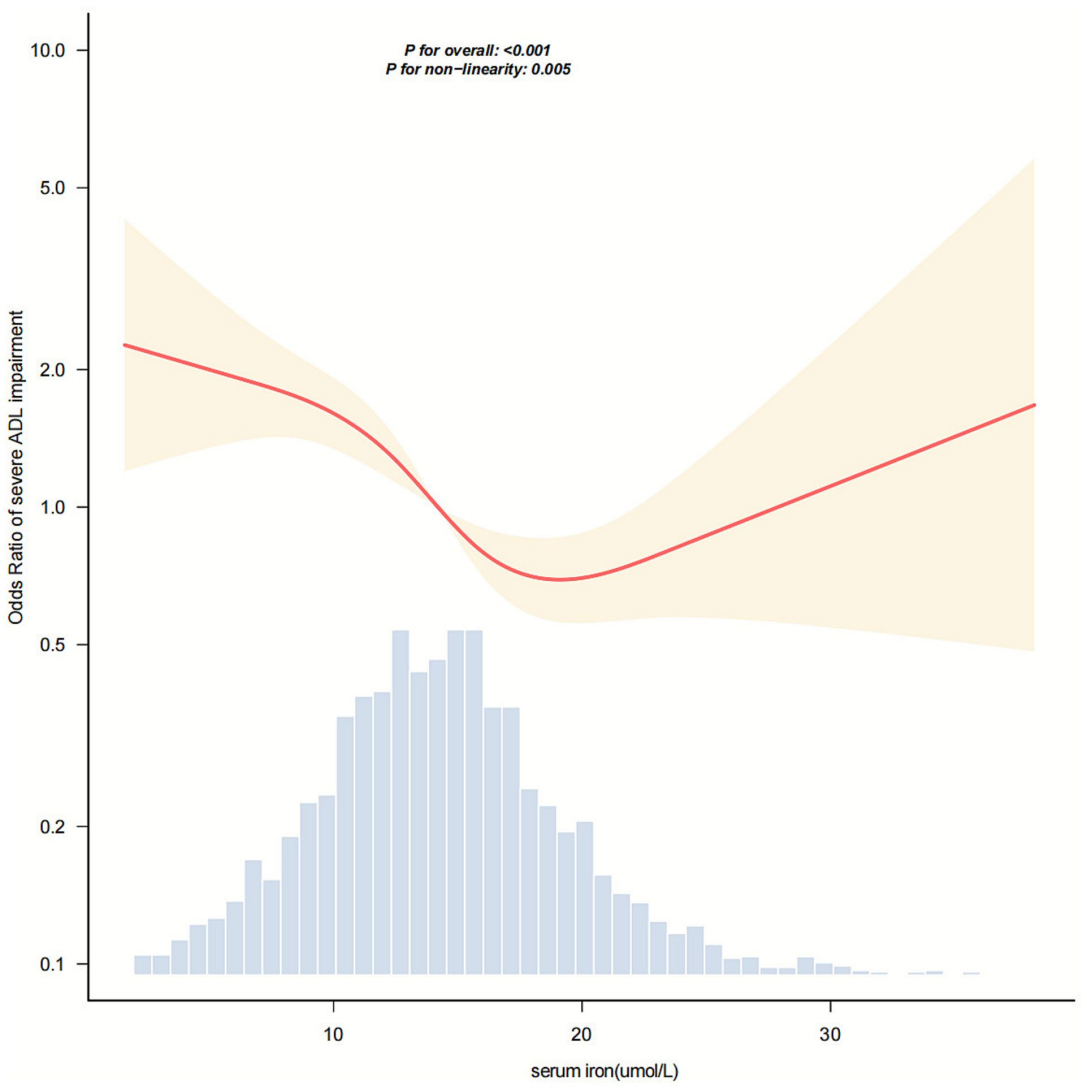
Dose–response relationship between serum iron levels and the odds of severe activities of daily living (ADL) impairment in stroke patients. The horizontal axis represents serum iron concentration (μmol/L), and the vertical axis represents the odds ratio for severe ADL impairment. The solid and dashed lines correspond to the predicted values and 95% confidence intervals, respectively. The model was adjusted for sex, age, BMI, smoking status, drinking status, hypertension, diabetes, coronary heart disease, stroke history, NIHSS score, TOAST classification, ALT, CRP, and ferritin levels. To facilitate visualization of the central trend, 99.8% of the data are shown after excluding extreme outliers.

In Figure 2, the relationship between serum iron levels and severe ADL impairment after stroke showed a nonlinear association (*p* = 0.005). The analysis identified an inflection point at approximately 17.5 μmol/L. In the threshold analysis, the odds ratio (OR) for severe ADL impairment among stroke patients with serum iron levels below 17.5 μmol/L was 0.91 (95% confidence interval [CI]: 0.876–0.946). Notably, when serum iron levels reached or exceeded 17.5 μmol/L, the association between serum iron and severe ADL impairment was no longer evident (Table 3).

**Table 3:** Threshold effect analysis of the relationship between serum iron and severe impairment of ADL in ischemic stroke patients.

Serum iron (μmol/L)	Adjusted model	
	OR (95% CI)	*p* value
<17.5	0.91 (0.876 ~ 0.946)	<0.001
≥17.5	1.021 (0.946 ~ 1.102)	0.601
Likelihood ratio test		0.018

OR, odds ratio; %, weighted proportion; CI, confidence interval.

Adjusted for sex, age, BMI, smoking status, and drinking status, hypertension, diabetes, coronary heart disease, and history of stroke, NIHSS, TOAST, ALT, CRP, and ferritin. Only 99.8% of the data is shown after excluding extremes outliers.

### Subgroup analyses

**Figure 3:**
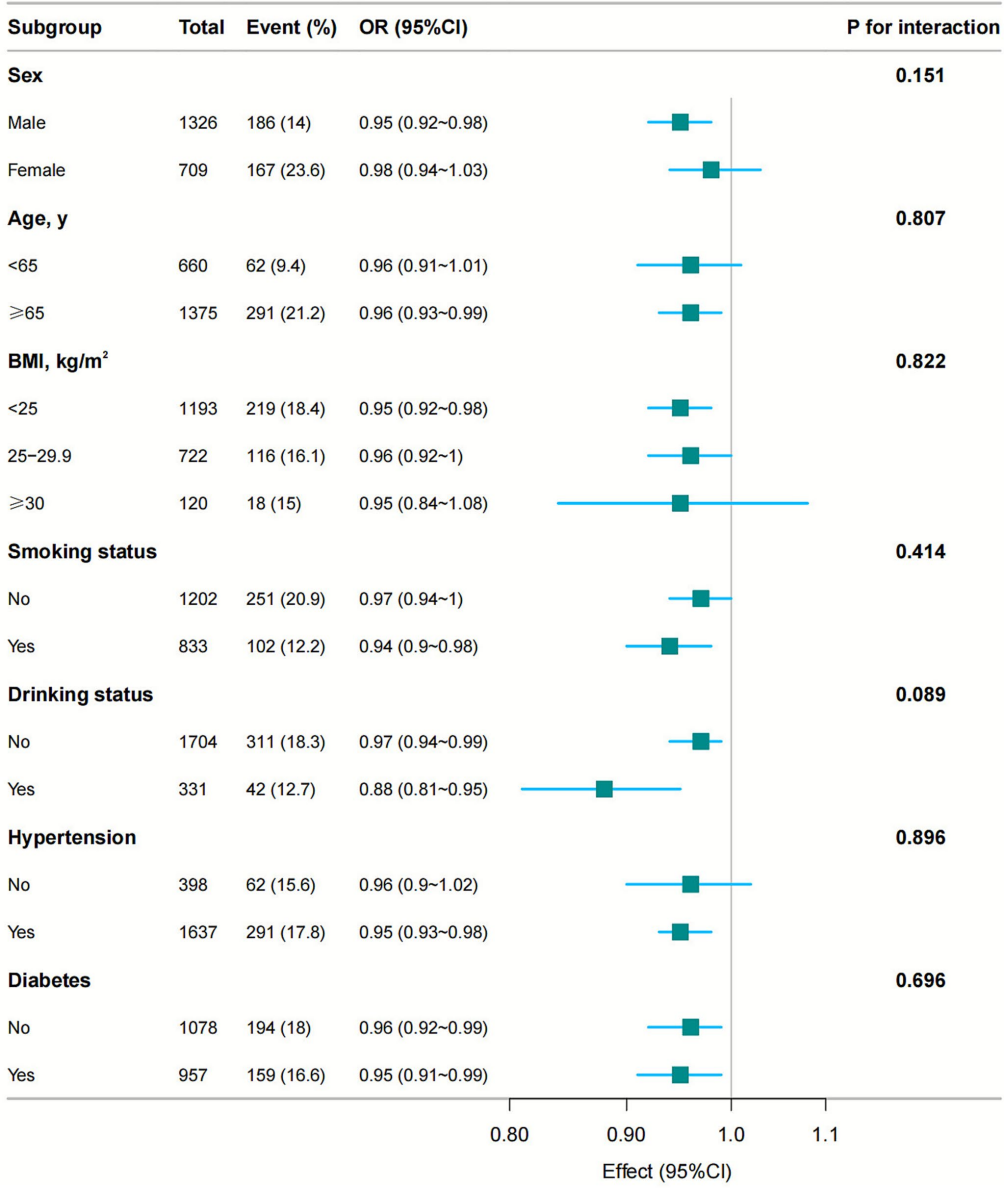
Association between serum iron levels and the odds ratio of severe ADL impairment in stroke patients, stratified by sex, age, BMI, smoking status, drinking status, hypertension, and diabetes. Each model was adjusted for all covariates, including coronary heart disease, stroke history, NIHSS score, TOAST classification, ALT, CRP, and ferritin levels.

Figure 3 presents the results of the subgroup analyses. Serum iron levels were significantly associated with impaired ability to perform activities of daily living in several subgroups, including males (OR, 0.95; 95% CI, 0.92–0.98), individuals aged over 65 years (OR, 0.96; 95% CI, 0.93–0.99), those with a BMI less than 25 kg/m^2^ (OR, 0.95; 95% CI, 0.92–0.98), smokers (OR, 0.94; 95% CI, 0.90–0.98), drinkers (OR, 0.88; 95% CI, 0.81–0.95), non-drinkers (OR, 0.97; 95% CI, 0.94–0.99), individuals with hypertension (OR, 0.95; 95% CI, 0.93–0.98), those with diabetes (OR, 0.95; 95% CI, 0.91–0.99), and individuals without diabetes (OR, 0.96; 95% CI, 0.92–0.99). No significant associations were observed in females under 65, individuals with BMI between 25–29.9 kg/m^2^ or ≥30 kg/m^2^, or among non-smokers and individuals without hypertension. Subgroup analyses based on sex, age, BMI, smoking, drinking, hypertension, and diabetes revealed no significant interactions (all *p*-values for interaction > 0.05).

### Sensitivity analysis

To address missing data, we applied multiple imputation across the entire study population. After adjusting for potential confounders, multivariate analysis showed that a 5 μmol/L increase in serum iron was associated with 26% lower odds of ADL impairment following stroke. Compared to individuals with lower serum iron levels (<11.1 μmol/L), the adjusted odds ratios (ORs) for severe ADL impairment in the second quartile (Q2: 11.1–14.2 μmol/L), third quartile (Q3: 14.2–17.3 μmol/L), and fourth quartile (Q4: >17.3 μmol/L) were 0.65 (95% confidence interval [CI]: 0.49–0.88), 0.45 (95% CI: 0.32–0.63), and 0.47 (95% CI: 0.33–0.68), respectively (Supplementary Table S3). Sensitivity analysis using a Barthel Index (BI) score of less than 60 as the cutoff for ADL dependence revealed a stronger and more consistent association between serum iron levels and ADL impairment after ischemic stroke (Supplementary Table S4). In this analysis, each 5-μmol/L increase in serum iron was associated with lower odds of severe ADL disability (OR = 0.82, 95% CI: 0.72–0.94), with an E-value of 1.74. In the categorical analysis, the E-values for Q2, Q3, and Q4 versus Q1 were 2.30, 4.08, and 3.11, respectively. Detailed results are provided in Supplementary Table S5.

## Discussion

Serum iron is a vital trace element involved in oxygen transport, cellular respiration, and antioxidant defense. Iron deficiency (ID) occurs when iron levels are insufficient to meet physiological needs (31) and has been linked to decreased physical performance and quality of life in adults, as well as cognitive decline in older individuals (32, 33). In this cross-sectional study of patients with ischemic stroke, we found a non-linear association between serum iron levels and severe ADL impairment in activities of daily living, with an inflection point at 17.5 μmol/L. No significant interactions were observed across subgroups divided by sex, age, BMI, smoking, alcohol consumption, hypertension, or diabetes. Sensitivity analyses supported the robustness of the findings.

Iron is essential for oxygen transport, mitochondrial energy production, antioxidant defense, DNA repair, neurotransmitter synthesis, and maintaining muscle function (7, 31). Disruptions in iron metabolism can affect neurological recovery after stroke. ID can impair mitochondrial function, antioxidant capacity, neurotransmitter production, and myelination, which can reduce neural plasticity and limit post-stroke repair. It may also lead to cognitive decline and muscle weakness (21, 34, 35). These biological mechanisms may explain the inverse association observed between serum iron and severe ADL impairment in our study, particularly as serum iron levels gradually increase. Conversely, iron overload leads to oxidative stress, endothelial dysfunction, blood–brain barrier disruption, and inflammatory responses involving cytokines such as IL-6 and TNF-*α*, thereby exacerbating ischemic injury (36–38). Excess iron may also increase blood viscosity, alter platelet function, and promote thrombogenesis (39, 40), all of which have been linked to ischemic stroke. Notably, no iron overload phenomenon was observed in this study, as we did not see an increase in severe ADL limitations following an increase in serum iron. This further validates the biological rationale behind the non-linear association. Once iron transporters and cellular uptake mechanisms reach saturation, excess iron does not enhance metabolic or neurodegenerative pathways beyond baseline requirements.

Growing evidence highlights the significance of iron metabolism in determining stroke outcomes. Elevated serum iron levels have been observed in patients with acute hemorrhagic stroke and may worsen neuronal injury through oxidative stress (41). Conversely, ID has been associated with poor functional recovery in acute stroke and increased long-term all-cause mortality among stroke survivors (35). Mendelian randomization studies suggest that although iron status may initially seem protective against large artery stroke or coronary heart disease, these associations become nonsignificant after adjusting for cardiovascular risk factors, indicating that iron status may be a modifiable factor for cardioembolic stroke (42). Similarly, relationships between ferritin levels and cardiovascular events disappear after multivariable adjustment (43). Recent multicenter cohort data further suggest that ID is an independent predictor of adverse 90-day functional outcomes in acute ischemic stroke (18).

The main strength of this study lies in its innovative examination of the association between serum iron levels and severe ADL impairment following ischemic stroke, including an analysis of the dose–response relationship.

However, several significant limitations must be acknowledged. First, serum iron levels were collected in the morning after an overnight fast to minimize diurnal variation; however, serum iron has a short half-life (44) and is acutely influenced by inflammation and disease severity. Although we adjusted for CRP and ferritin in multivariate models, residual confounding cannot be fully ruled out. We did not systematically measure transferrin saturation or hepcidin, preventing a comprehensive assessment of iron metabolism. Future studies should incorporate multiple iron metabolism indicators measured serially to characterize iron homeostasis better.

Second, despite regression modeling and sensitivity analyses, unmeasured confounding cannot be entirely discounted. Essential factors, such as anemia status, nutritional indices, renal function, stroke volume, large vessel occlusion, and reperfusion therapy, were not fully captured. E-value calculations suggested that substantial unmeasured confounding would be required to explain the observed associations fully; however, residual confounding remains a possibility.

Third, 37% of patients were excluded due to missing serum iron data, which may introduce selection bias. We performed multiple imputation and sensitivity analyses to assess the robustness of our results. Additionally, pre-admission functional status could not be ascertained, making it challenging to distinguish baseline impairment from stroke-related ADL disability. A history of prior stroke was included as a proxy, but may not fully capture pre-existing limitations. Future studies should prospectively collect baseline functional status data to isolate stroke-attributable ADL impairment better.

Fourth, and most critically, the cross-sectional design fundamentally precludes establishing causality between serum iron levels and severe ADL impairment. Our findings reflect associations only and cannot determine whether low serum iron contributes to poor outcomes or is merely a consequence of stroke severity and inflammation.

To establish causality, future prospective or intervention studies are needed to examine whether interventions targeting iron homeostasis can improve functional recovery, while controlling for confounders such as inflammation, anemia, nutritional status, and stroke severity.

## Conclusion

Our study found a non-linear relationship between serum iron levels and severe ADL impairment in ischemic stroke patients, with an inflection point at approximately 17.5 μmol/L. Given the cross-sectional design and limited available information, these findings should be interpreted with caution.

## Data Availability

The original contributions presented in the study are included in the article/Supplementary material, further inquiries can be directed to the corresponding author.

## References

[1] Feigin VL Owolabi MO. Pragmatic solutions to reduce the global burden of stroke: a world stroke organization-lancet neurology commission. Lancet Neurol. (2023) 22:1160–206. doi: 10.1016/s1474-4422(23)00277-6, 37827183 PMC10715732

[2] Huang M Xu S Zhou M Luo J Zha F Shan L . Lysophosphatidylcholines and phosphatidylcholines as biomarkers for stroke recovery. Front Neurol. (2022) 13:1047101. doi: 10.3389/fneur.2022.1047101, 36588912 PMC9797831

[3] Feigin VL Brainin M Norrving B Martins SO Pandian J Lindsay P . World stroke organization: global stroke fact sheet 2025. Int J Stroke. (2025) 20:132–44. doi: 10.1177/17474930241308142, 39635884 PMC11786524

[4] Prust ML Forman R Ovbiagele B. Addressing disparities in the global epidemiology of stroke. Nat Rev Neurol. (2024) 20:207–21. doi: 10.1038/s41582-023-00921-z, 38228908

[5] Tu WJ Wang LD. China stroke surveillance report 2021. Mil Med Res. (2023) 10:33. doi: 10.1186/s40779-023-00463-x, 37468952 PMC10355019

[6] Zhou Y Zhang S Fan X. Role of polyphenols as antioxidant supplementation in ischemic stroke. Oxidative Med Cell Longev. (2021) 2021:5471347. doi: 10.1155/2021/5471347, 34257802 PMC8253632

[7] Azzollini V Dalise S Chisari C. How does stroke affect skeletal muscle? State of the art and rehabilitation perspective. Front Neurol. (2021) 12:797559. doi: 10.3389/fneur.2021.797559, 35002937 PMC8733480

[8] Weber RZ Achon Buil B Rentsch NH Perron P Halliday S Bosworth A . Neural xenografts contribute to long-term recovery in stroke via molecular graft-host crosstalk. Nat Commun. (2025) 16:8224. doi: 10.1038/s41467-025-63725-3, 40957886 PMC12441158

[9] Somerville E Blenden G Kretzer D Holden B Bollinger RM Krauss MJ . Differences in daily activity performance between inpatient rehabilitation facility and home among stroke survivors. Neurorehabil Neural Repair. (2024) 38:403–12. doi: 10.1177/15459683241246266, 38602200 PMC11100317

[10] Ozkan H Ambler G Banerjee G Mitchell JJ Barbato C Browning S . Prevalence, predictors, and patterns of patient reported non-motor outcomes six months after stroke: a prospective cohort study. Lancet Reg Health Eur. (2024) 47:101080. doi: 10.1016/j.lanepe.2024.101080, 39498117 PMC11532962

[11] Zeng S Wu M Xu L Guo Z Chen S Ling K . Challenges in accessing community-based rehabilitation and long-term care for older adult stroke survivors and their caregivers: a qualitative study. J Multidiscip Healthc. (2024) 17:4829–38. doi: 10.2147/jmdh.S476993, 39464787 PMC11512762

[12] Huang L Li W Lu Y Ju Q Ouyang M. Iron metabolism in colorectal cancer. Front Oncol. (2023) 13:1098501. doi: 10.3389/fonc.2023.1098501, 36910614 PMC9992732

[13] Bi Y Ajoolabady A Demillard LJ Yu W Hilaire ML Zhang Y . Dysregulation of iron metabolism in cardiovascular diseases: from iron deficiency to iron overload. Biochem Pharmacol. (2021) 190:114661. doi: 10.1016/j.bcp.2021.114661, 34157296

[14] Grubić Kezele T Ćurko-Cofek B. Age-related changes and sex-related differences in brain Iron metabolism. Nutrients. (2020) 12:2601. doi: 10.3390/nu12092601, 32867052 PMC7551829

[15] Chen L Shen Q Liu Y Zhang Y Sun L Ma X . Homeostasis and metabolism of iron and other metal ions in neurodegenerative diseases. Signal Transduct Target Ther. (2025) 10:31. doi: 10.1038/s41392-024-02071-0, 39894843 PMC11788444

[16] He Q Wang W Xu D Xiong Y You C Tao C . Causal Association of Iron Status with Functional Outcome after Ischemic Stroke. Stroke. (2024) 55:423–31. doi: 10.1161/strokeaha.123.044930, 38095120

[17] Xia X Liu J Fang W Chen Z Wang J Xu H. The association between ferritin levels and all-cause mortality in stroke patients. Front Neurol. (2024) 15:1386408. doi: 10.3389/fneur.2024.1386408, 38988599 PMC11233758

[18] Ciacciarelli A Falcou A Nicolini E Broccolini A Frisullo G Abruzzese S . The prognostic role of iron deficiency in acute ischemic stroke patients: a prospective multicentric cohort study. J Neurol Sci. (2025) 469:123371. doi: 10.1016/j.jns.2024.123371, 39764913

[19] Wu Q Wei C Liu J Wang Y Liu M. Effects of Hyperferritinemia on functional outcome in acute ischemic stroke patients with admission hyperglycemia. Cerebrovasc Dis. (2023) 52:511–8. doi: 10.1159/000527860, 36516789

[20] Zheng W Wang Y Xia Z Liu D. Serum ferritin and risk of stroke: a meta-analysis of observation studies. Front Neurol. (2025) 16:1539407. doi: 10.3389/fneur.2025.1539407, 40667468 PMC12259440

[21] Claus JJ Berghout BBP Ikram MK Wolters FJ. Validity of stroke severity assessment using medical records in a population-based cohort. J Stroke Cerebrovasc Dis. (2023) 32:106992. doi: 10.1016/j.jstrokecerebrovasdis.2023.106992, 36801651

[22] Comer AR Templeton E Glidden M Bartlett S D’Cruz L Nemati D . National Institutes of Health stroke scale (NIHSS) scoring inconsistencies between neurologists and emergency room nurses. Front Neurol. (2022) 13:1093392. doi: 10.3389/fneur.2022.1093392, 36712449 PMC9875120

[23] Langanay L Gonzalez Sanchez R Hamroun A Dauchet L Amouyel P Dallongeville J . Ischemic stroke subtypes: risk factors, treatments, and 1-month prognosis - the Lille, France stroke registry. J Stroke Cerebrovasc Dis. (2024) 33:107761. doi: 10.1016/j.jstrokecerebrovasdis.2024.107761, 38723923

[24] Mahoney FI Barthel DW. Functional evaluation: the Barthel index. Md State Med J. (1965) 14:61–5. 14258950

[25] Wang E Liu A Wang Z Shang X Zhang L Jin Y . The prognostic value of the Barthel index for mortality in patients with COVID-19: a cross-sectional study. Front Public Health. (2022) 10:978237. doi: 10.3389/fpubh.2022.978237 36761326 PMC9902915

[26] Bi R Shi Y Li M Liu X Ma Z Huang Y . Association between serum albumin and severe impairment of activities of daily living in patients with stroke: a cross-sectional study. Front Neurol. (2024) 15:1501294. doi: 10.3389/fneur.2024.1501294, 39835151 PMC11743378

[27] Wasiak K Frasuńska J Tarnacka B. Can the initial parameters of functional scales predict recovery in patients with complete spinal cord injury? A retrospective cohort study. Diagnostics. (2024) 14:129. doi: 10.3390/diagnostics14020129, 38248006 PMC10814489

[28] Yi Y Ding L Wen H Wu J Makimoto K Liao X. Is Barthel index suitable for assessing activities of daily living in patients with dementia? Front Psych. (2020) 11:282. doi: 10.3389/fpsyt.2020.00282, 32457659 PMC7225343

[29] López-Ortiz S Valenzuela PL Seisdedos MM Morales JS Vega T Castillo-García A . Exercise interventions in Alzheimer’s disease: a systematic review and meta-analysis of randomized controlled trials. Ageing Res Rev. (2021) 72:101479. doi: 10.1016/j.arr.2021.101479, 34601135

[30] Granger CV Dewis LS Peters NC Sherwood CC Barrett JE. Stroke rehabilitation: analysis of repeated Barthel index measures. Arch Phys Med Rehabil. (1979) 60:14–7. 420565

[31] Iolascon A Andolfo I Russo R Sanchez M Busti F Swinkels D . Recommendations for diagnosis, treatment, and prevention of iron deficiency and iron deficiency anemia. Hemasphere. (2024) 8:e108. doi: 10.1002/hem3.108, 39011129 PMC11247274

[32] Peng J Liu B Tan W Hu S Li B Zhou J . Association between body Iron status and cognitive task performance in a nationally representative sample of older adults. Aging Dis. (2024) 16:1141–8. doi: 10.14336/ad.2019.0064, 38739935 PMC11964420

[33] Gingoyon A Borkhoff CM Koroshegyi C Mamak E Birken CS Maguire JL . Chronic Iron deficiency and cognitive function in early childhood. Pediatrics. (2022) 150:e2021055926. doi: 10.1542/peds.2021-055926, 36412051

[34] Doehner W Scherbakov N Schellenberg T Jankowska EA Scheitz JF von Haehling S . Iron deficiency is related to low functional outcome in patients at early rehabilitation after acute stroke. J Cachexia Sarcopenia Muscle. (2022) 13:1036–44. doi: 10.1002/jcsm.12927, 35166066 PMC8977949

[35] Wang Q Han W Wang T Deng H Zhong J. The impact of iron deficiency on prognosis in stroke and non-stroke populations: a retrospective cohort study. Nutr Metab Cardiovasc Dis. (2025) 35:103759. doi: 10.1016/j.numecd.2024.09.029, 39571324

[36] Obeagu EI Obeagu GU. Anemia and cerebrovascular disease: pathophysiological insights and clinical implications. Ann Med Surg. (2025) 87:3254–3267. doi: 10.1097/ms9.0000000000002907, 40486649 PMC12140788

[37] Jomova K Alomar SY Valko R Nepovimova E Kuca K Valko M. The role of redox-active iron, copper, manganese, and redox-inactive zinc in toxicity, oxidative stress, and human diseases. EXCLI J. (2025) 24:880–954. doi: 10.17179/excli2025-8449, 40933952 PMC12419454

[38] Rosenblum SL. Inflammation, dysregulated iron metabolism, and cardiovascular disease. Front Aging. (2023) 4:1124178. doi: 10.3389/fragi.2023.1124178, 36816471 PMC9935942

[39] Brissot E Troadec MB Loréal O Brissot P. Iron and platelets: a subtle, under-recognized relationship. Am J Hematol. (2021) 96:1008–16. doi: 10.1002/ajh.26189, 33844865

[40] Liu M Chen M Hao Z Li Q Feng Y Li Y . Erythrocyte fraction in thrombi is increased with serum Iron by influencing fibrin networks via oxidative stress. Oxidative Med Cell Longev. (2021) 2021:3673313. doi: 10.1155/2021/3673313, 34976298 PMC8719990

[41] Hao L Zhang A Lv D Gao M Guo W Yao Z. Exploring the link between iron dysregulation, ferroptosis, and cognitive dysfunction in intracerebral hemorrhage patients. J Clin Neurosci. (2025) 135:111194. doi: 10.1016/j.jocn.2025.111194, 40132332

[42] Barad A Clark AG Pressman EK O’Brien KO. Associations between genetically predicted iron status and cardiovascular disease risk: a Mendelian randomization study. J Am Heart Assoc. (2024) 13:e034991. doi: 10.1161/jaha.124.034991, 38818967 PMC11255641

[43] Quintana Pacheco DA Sookthai D Wittenbecher C Graf ME Schübel R Johnson T . Red meat consumption and risk of cardiovascular diseases-is increased iron load a possible link? Am J Clin Nutr. (2018) 107:113–9. doi: 10.1093/ajcn/nqx014, 29381787

[44] Nguyen LT Buse JD Baskin L Sadrzadeh SMH Naugler C. Influence of diurnal variation and fasting on serum iron concentrations in a community-based population. Clin Biochem. (2017) 50:1237–42. doi: 10.1016/j.clinbiochem.2017.09.018, 28947322

