# Supplementary material for: The non-linear association between serum iron and severe impairment of activities of daily living in ischemic stroke patients
Source: Front Neurol. 2026 Jan 15;16:1700381. doi: 10.3389/fneur.2025.1700381 (PMC12852888; doi:10.3389/fneur.2025.1700381)
Supplement: Supplementary file 1 [file Table_1.docx]

**Table S1.** The missing number and rate of covariates for baseline analyses.

| **Variables** | **Total  (n=** 2035**)** |
| --- | --- |
| Age | 0(0.00%) |
| Gender | 0(0.00%) |
| BMI | 296(14.50%) |
| Smoking status | 175(8.60%) |
| Drinking status | 85(4.18%) |
| Hypertension | 34(1.67%) |
| Diabetes | 34(1.67%) |
| Coronary heart disease | 34(1.67%) |
| Atrial fibrillation | 34(1.67%) |
| Cancer | 34(1.67%) |
| History of stroke | 34(1.67%) |
| TOAST | 255(12.50%) |
| NIHSS | 475(23.30%) |
| ALT | 81(3.98%) |
| CPR | 307(15.09%) |
| Ferritin | 950(46.68%) |

Abbreviations: BMI, body mass index; TOAST: Trial of Org 10172 in Acute Stroke Treatment; NIHSS: National Institutes of Health Stroke Scale; ALT: Alanine Aminotransferase; CRP: C-reactive Protein.

**Table S2. Characteristics of included and excluded participants for missing data at baseline.**

| Variables | Total  (n = 3365) | Include  (n = 2035) | Exclude  (n = 1330) | *P* value |
| --- | --- | --- | --- | --- |
| Gender, n (%) |  |  |  | 0.053 |
| Male | 2149 (63.9) | 1326 (65.2) | 823 (61.9) |  |
| Female | 1216 (36.1) | 709 (34.8) | 507 (38.1) |  |
| Age, Mean ± SD | 69.5 ± 11.6 | 69.3 ± 11.7 | 69.7 ± 11.5 | 0.289 |
| BMI, Mean ± SD | 24.6 ± 3.4 | 24.5 ± 3.4 | 24.6 ± 3.4 | 0.473 |
| Smoking status, n (%) |  |  |  | 0.016 |
| No | 1883 (60.2) | 1088 (58.5) | 795 (62.8) |  |
| Yes | 1243 (39.8) | 772 (41.5) | 471 (37.2) |  |
| Drinking status, n (%) |  |  |  | < 0.001 |
| No | 2567 (79.9) | 1637 (83.9) | 930 (73.7) |  |
| Yes | 645 (20.1) | 313 (16.1) | 332 (26.3) |  |
| Hypertension, n (%) |  |  |  | 0.017 |
| No | 699 (21.3) | 398 (19.9) | 301 (23.4) |  |
| Yes | 2589 (78.7) | 1603 (80.1) | 986 (76.6) |  |
| Diabetes, n (%) |  |  |  | < 0.001 |
| No | 1848 (56.2) | 1062 (53.1) | 786 (61.1) |  |
| Yes | 1440 (43.8) | 939 (46.9) | 501 (38.9) |  |
| Coronary heart disease, n (%) |  |  |  | 0.395 |
| No | 2751 (83.7) | 1683 (84.1) | 1068 (83) |  |
| Yes | 537 (16.3) | 318 (15.9) | 219 (17) |  |
| Atrial fibrillation, n (%) | |  |  | 0.353 |
| No | 2917 (88.7) | 1767 (88.3) | 1150 (89.4) |  |
| Yes | 371 (11.3) | 234 (11.7) | 137 (10.6) |  |
| Cancer, n (%) |  |  |  | 0.516 |
| No | 2913 (88.6) | 1767 (88.3) | 1146 (89) |  |
| Yes | 375 (11.4) | 234 (11.7) | 141 (11) |  |
| History of stroke, n (%) | |  |  | < 0.001 |
| No | 2785 (84.7) | 1643 (82.1) | 1142 (88.7) |  |
| Yes | 503 (15.3) | 358 (17.9) | 145 (11.3) |  |
| TOAST, n (%) |  |  |  | < 0.001 |
| large-artery atherosclerosis | 1203 (45.3) | 715 (40.2) | 488 (55.8) |  |
| small-vessel occlusion | 968 (36.5) | 736 (41.3) | 232 (26.5) |  |
| cardio embolism | 240 (9.0) | 174 (9.8) | 66 (7.5) |  |
| stroke of another determined etiology | 118 (4.4) | 42 (2.4) | 76 (8.7) |  |
| stroke of undetermined etiology | 126 (4.7) | 113 (6.3) | 13 (1.5) |  |
| NIHSS, Median (IQR) | 2.0 (1.0, 5.0) | 2.0 (1.0, 5.0) | 2.0 (1.0, 5.0) | 0.015 |
| Fe, Mean ± SD | 14.3 ± 6.2 | 14.4 ± 6.1 | 11.5 ± 6.5 | < 0.001 |
| ALT, Median (IQR) | 16.0 (11.0, 24.0) | 15.0 (11.0, 22.0) | 22.0 (15.0, 32.0) | < 0.001 |
| CRP, Median (IQR) | 5.8 (2.6, 16.4) | 6.9 (3.0, 19.0) | 4.3 (1.9, 12.0) | < 0.001 |
| Ferritin, Median (IQR) | 233.0 (138.8, 371.0) | 228.0 (138.0, 362.0) | 241.5 (140.0, 391.0) | 0.334 |

**Table S3. Association between serum albumin and impairment of ADL in Ischemic stroke patients after multiple imputation (All patients)**

| Variable | n. total | n. event_% | Crude model | | Model 1 | | Model 2 | | Model 3 | |
| --- | --- | --- | --- | --- | --- | --- | --- | --- | --- | --- |
|  |  |  | OR（95%CIs） | *P* value | OR（95%CIs） | *P* value | OR（95%CIs） | *P* value | OR（95%CIs） | *P* value |
| serum iron (5µmol/L) | 3365 | 578 (17.2) | 0.48 (0.44~0.54) | <0.001 | 0.55 (0.49~0.62) | <0.001 | 0.69 (0.61~0.78) | <0.001 | 0.74 (0.66~0.84) | <0.001 |
| serum iron Group (µmol/L) |  |  |  |  |  |  |  |  |  |  |
| Q1（＜11.1） | 840 | 294 (35) | 1(Ref) |  | 1(Ref) |  | 1(Ref) |  | 1(Ref) |  |
| Q2（11.1-14.2） | 841 | 133 (15.8) | 0.35 (0.28~0.44) | <0.001 | 0.39 (0.31~0.5) | <0.001 | 0.56 (0.42~0.74) | <0.001 | 0.65 (0.49~0.88) | 0.005 |
| Q3（14.2-17.3） | 840 | 78 (9.3) | 0.19 (0.14~0.25) | <0.001 | 0.24 (0.18~0.31) | <0.001 | 0.39 (0.28~0.54) | <0.001 | 0.45 (0.32~0.63) | <0.001 |
| Q4（≥17.3） | 844 | 73 (8.6) | 0.18 (0.13~0.23) | <0.001 | 0.24 (0.18~0.32) | <0.001 | 0.4 (0.29~0.56) | <0.001 | 0.47 (0.33~0.68) | <0.001 |
| P for Trend |  |  |  | <0.001 |  | <0.001 |  | <0.001 |  | <0.001 |

Q, quartiles; OR, odds ratio; CI, confidence interval; Ref: reference.

Crude model: No adjustment.

Model 1 was adjusted for sex, age, BMI, smoking status, and drinking status.

Model 2 was adjusted for sex, age, BMI, smoking status, drinking status, hypertension, diabetes, coronary heart disease, history of stroke, NIHSS, and TOAST.

Model 3 was adjusted for sex, age, BMI, smoking status, drinking status, hypertension, diabetes, coronary heart disease, history of stroke, NIHSS, TOAST, ALT, CRP, and ferritin.

**Table S4. Association between serum albumin and impairment of ADL in ischemic stroke patients after multiple imputation (ADL grouped by 60 scores)**

| Variable | n. total | n. event_% | Crude model | | Model 1 | | Model 2 | | Model 3 | |
| --- | --- | --- | --- | --- | --- | --- | --- | --- | --- | --- |
|  |  |  | OR（95%CIs） | *P* value | OR（95%CIs） | *P* value | OR（95%CIs） | *P* value | OR（95%CIs） | *P* value |
| serum iron (5µmol/L) | 2035 | 840 (41.3) | 0.73 (0.67~0.79) | <0.001 | 0.79 (0.73~0.86) | <0.001 | 0.88 (0.8~0.96) | 0.004 | 0.91 (0.83~0.99) | 0.034 |
| serum iron Group (µmol/L) |  |  |  |  |  |  |  |  |  |  |
| Q1（＜10.3） | 504 | 292 (57.9) | 1(Ref) |  | 1(Ref) |  | 1(Ref) |  | 1(Ref) |  |
| Q2（10.3-13.9） | 513 | 215 (41.9) | 0.52 (0.41~0.67) | <0.001 | 0.6 (0.46~0.77) | <0.001 | 0.79 (0.59~1.06) | 0.11 | 0.85 (0.64~1.15) | 0.297 |
| Q3（13.9-18.0） | 508 | 178 (35) | 0.39 (0.3~0.5) | <0.001 | 0.47 (0.36~0.61) | <0.001 | 0.66 (0.49~0.89) | 0.007 | 0.72 (0.53~0.97) | 0.034 |
| Q4（≥18.0） | 510 | 155 (30.4) | 0.32 (0.24~0.41) | <0.001 | 0.41 (0.31~0.54) | <0.001 | 0.6 (0.44~0.81) | 0.001 | 0.66 (0.48~0.9) | 0.009 |
| P for Trend |  |  |  | <0.001 |  | <0.001 |  | <0.001 |  | 0.005 |

Q, quartiles; OR, odds ratio; CI, confidence interval; Ref: reference.

Crude model: No adjustment.

Model 1 was adjusted for sex, age, BMI, smoking status, and drinking status.

Model 2 was adjusted for sex, age, BMI, smoking status, drinking status, hypertension, diabetes, coronary heart disease, history of stroke, NIHSS, and TOAST.

Model 3 was adjusted for sex, age, BMI, smoking status, drinking status, hypertension, diabetes, coronary heart disease, history of stroke, NIHSS, TOAST, ALT, CRP, and ferritin.

**Table S5. E-values for serum albumin and severe impairment of ADL in Ischemic stroke patients**

| Variable | OR | 95% CI | E-value (Estimate) |
| --- | --- | --- | --- |
| serum iron (5µmol/L) | 0.82 | 0.72~0.94 | 1.74 |
| serum iron Group (µmol/L) |  |  |  |
| Q1（＜10.3） | 1 | -- | -- |
| Q2（10.3-13.9） | 0.68 | 0.47~1 | 2.30 |
| Q3（13.9-18.0） | 0.43 | 0.28~0.66 | 4.08 |
| Q4（≥18.0） | 0.54 | 0.35~0.83 | 3.11 |

Q, quartiles; OR, odds ratio; CI, confidence interval; Ref: reference.
